# Influence of hollow droplet impact and spreading characteristics on the cylindrical lateral surface

**DOI:** 10.1016/j.heliyon.2024.e38320

**Published:** 2024-09-23

**Authors:** Hossein Sayyari, Mohammad Mohsen Peiravi, Javad Alinejad

**Affiliations:** a Department of Mechanical Engineering, Sari Branch, Islamic Azad University, Sari, Iran; b Department of Mechanical Engineering, Babol Noshirvani University of Technology, Babol, Iran

**Keywords:** Hollow droplet impact, Hydrodynamic behavior, Spreading characteristics, VOF method

## Abstract

In this study, 3D model of a hollow glycerin droplet impacting on a cylindrical cross-section was numerically analyzed to coat the lateral surface of the cylinder. The hollow droplets had exterior diameters of 5.5 mm, 5.25 mm, and 5 mm, and the impact velocity was 1 m/s. To model, the influence of droplets on OpenFoam software, the Volume Of Fluid technique was utilized (VOF). The Newtonian, incompressible, and laminar fluid phase of a glycerin droplet was investigated using air with a diameter of 4 mm as the gas phase. The hydrodynamic behavior of droplet collisions as well as fluid properties were investigated. As a result, when can be observed in the simple cylinder, as the outer diameter increased, the spread diameter shrank. The least amount distributed diameter in Case 3 was 1.404, whereas the highest amount in Case 4 was 1.625. The situations with a simple lateral surface, uniform spread occurred, while in cases with a spiral lateral surface, non-uniform spread occurred. Observed the maximum length in Case 3 was 4.12 mm and the minimum was 1.83 mm in Case 4.

## 1 Introduction

As a response to the hollow drop colliding on various surfaces in industry or nature, spray coating, spray painting, drop of rain, plasma spray, and thermal spray coating are used. Droplet impact is influenced by dynamic behavior, impact processes, and dissemination variables. Experiments and analytical modeling have produced numerous works in dynamics on the impingement of a single droplet.

In order to manage the rate of heat transfer under various heat fluxes, experimentation and analysis methods were utilized to analyse 2 different nano - fluids natural convection within the reconfigurable inclosure [1]. Based on a dynamic contact angle of the drop at the spatial boundary between two substances, scholars have researched and breakup of droplets on 2 rectangular cylindrical [2]. To fully appreciate the fluid flow that occurs on the surface, scientists have studied the effect of the adhesion of drops on a surface [3]. The lattice Boltzmann technique is used to analyse forced convection through an electronic board fitted with numerous different forms of barriers, composed of 3 cylindrical and 3 cubes (LBM) [4]. The droplet behaviors of Newtonian and non-Newtonian droplets impinging on a heated surface are examined using three-dimensional computational fluid dynamics analysis. The liquid's free surface is also monitored using the volume-of-fluid (VOF) method. When evaluating the impact of viscosity, density, and surface tension on droplet dynamics, temperature dependency is taken into account [5]. Oil droplets lost from the bearing in a bearings chamber contact the outside chamber housing at various angles. The research presented in this paper serves as a basis for understanding the lubrication conditions in bearing chambers as well as a crucial source of information for the investigation of droplet-solid surface collisions [6]. Droplet impact on elastic beams is regarded as a unique model of energy transmission that offers a possible substitute in energy-harvesting applications. The fluid-solid contact and capillary effect are the dominant forces in the transient impact process. The super-hydrophobic (SH) surfaces can influence droplet dynamics, and the numerical model based on the SPH approach can predict these behaviors [7]. The researchers characterized and studied the specific phenomenon of "shock drop" in the dye-on-demand (DOD) process. A series of simulations based on a piezoelectric DOD print test system are presented, adapting the volume of fluid (VOF) interface capture method to follow the evolution of the boundary and model the interface physics [8]. Utilising a high-speed video recorder, the collision mechanism of a liquid droplet hitting with a revolving disc was captured and examined. To investigate their effects on the results of fluid flow, four disc contacting radii, eight rotating speeds, and four drop falling velocities were used. In fact, the radially spread ratio is significantly influenced by the drop's falling velocity while being barely affected by the Rossby number [9]. In order to make coating with combined nanomaterials in Silica and Titania sol matrix, sol-gel spray coatings was utilized. The coatings were examined using scanning electron microscopy (SEM), FT-IR spectroscopy, and infrared heat photography. The coatings have the potential to be employed in radiative cooling applications since they can be created using the sol-gel technique in a straightforward and regulated manner [10]. To model the collision of a droplet with sloped dry walls caused by gravitational force, the phase field approach of the Lattice Boltzmann method (LBM) is used. In order to confirm the convective boundary condition as the outlet one, this approach was initially used to explore the dynamic behavior of moving droplets in a horizontal channel [11]. The impact behavior of a water droplet on small cylindrical superhydrophobic targets was studied numerically and theoretically. A numerical model using the volume of liquid method is developed to simulate the impact process of droplets on small superhydrophobic cylindrical targets [12]. Droplet bounce is ubiquitous on super-resistant surfaces. The conversion between kinetic and surface energies suggests that annealing suppression is impossible due to negligible energy dissipation. Capillary flows induced by the deformed surface of the inner bubble offset those induced by the outer surface of the droplet, resulting in a reduction of the effective lifting moment [13].

The 3D model of a hollow glycerin drop colliding on a cylindrical cross-section to coat the lateral surface of the cylinder was used in this investigation. It was looked into the impact process, dynamic behavior, and fluid characteristics. This research work provides the fluid velocity and spreading factors of a three-dimensional model of a hollow droplet collision.

## 2 Problem definition

**Fig. 1:**
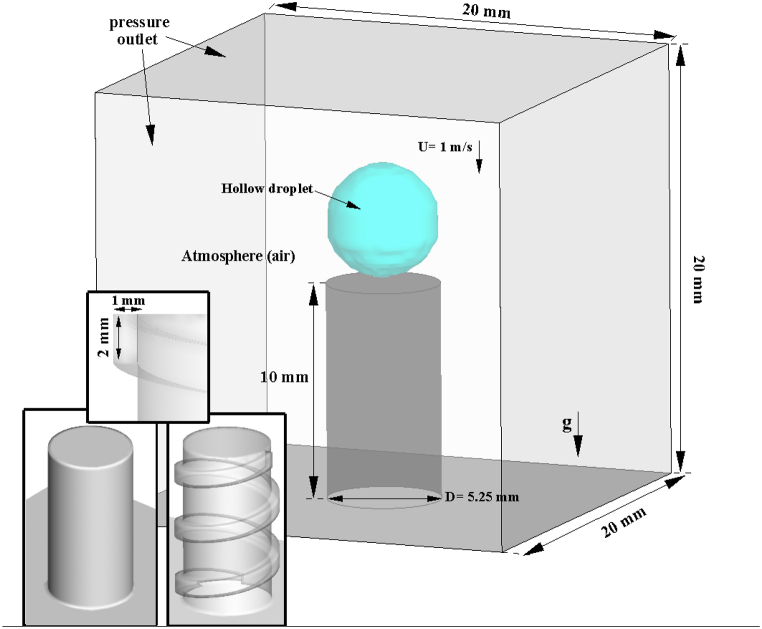
An illustration of a computation area.

In a 3D model, a hollow droplet has an impact on the cylindrical cross-section at various diameters. After impact, a hollow droplet spreads across the cylinder's lateral surface, which is simple and grooved. The hollow droplet hit the center point of the cylinder's cross-section at a velocity of 1 m/s. As shown in Fig. 1, the computational domain is a cube with a 20 mm diameter. The outside diameters of hollow droplets are 5.5 mm, 5.25 mm, and 5 mm, respectively, while the inner diameters are 4.25 mm, 4 mm, and 3.75 mm. The radius of the shell thickness was 0.625 mm. Glycerin droplets have a density of 1261 $kg/m^{3}$, a viscosity of 0.142 $kg.m^{-1}/s$ , and surface tension of 0.0634 N/m. The air cavity in the hollow droplet has a density of 1.225 $kg/m^{3}$ and a viscosity of $1.7894\times 10^{-5}kg.m^{-1}/s$. The droplets made contact with the surface at a 140° angle. The fluid is Newtonian, incompressible, and laminar [14,18]. Table .1 shows the case numbers, cylinder shape, and hollow droplet diameter.

**Table 1:** Dimension of hollow droplet diameter and shape of the cylinder for each case.

	Outer diameter (mm)	Inner diameter (mm)	Type of cylinder
Case1	5	3.75	Simple
Case2	5.25	4	Simple
Case3	5.5	4.25	Simple
Case4	5	3.75	Spiral
Case5	5.25	4	Spiral
Case6	5.5	4.25	Spiral

## 3 Simulation methodology

### 3.1 Volume of fluid

The study focused on the fluid volume approach applied to a three-dimensional model analyzing the impact of hollow drops on a cylindrical cross-section with varying diameters of the hollow drops. This fluid volume model is employed to calculate a series of momentum equations that track the boundary between the droplets and the surrounding atmosphere. The volume percentage of liquid, represented as β=1−φ, is considered to be (0<φ<1) in a control volume (***φ***). The environment of the hollow droplet interface is characterized by a value of φ=0, which signifies that the cell contains air, while a value of φ=1 indicates that the cell is filled with glycerin droplets. The time elapsed after impact is represented in equation (1). Two-phase contact flow exhibits unique facial characteristics. The continuum mixing velocity profile U is employed in the volume fraction indicator. This is addressed through the relevant transport equation:

(1) $$\frac{D\varphi }{D\tau }=\frac{\partial \varphi }{\partial \tau }+\left[\frac{\partial U_{x}}{\partial x}+\frac{\partial U_{y}}{\partial y}+\frac{\partial U_{z}}{\partial z}\right]\varphi =0$$

To produce the proper curvature at the contact, a precise estimate of volume status fraction dispersion is necessary. The below is just how Poo et al. [15] describe how a movement term is added to the volume concentration dispersion equation [16].

(2) $$\frac{\partial \varphi }{\partial \tau }+\left(\varphi \nabla .U+U.\nabla \varphi \right)+\nabla .\left(\varphi \beta U_{c}\right)=0$$

Compress at the interface is created by the last clause of equation (2), changing the volume percentage and boundary of each phase. Equations (3), (4) states that $U_{c}$ is the required velocity for effective compressing at the interface.

(3) $$U_{c}=\min\left[C_{\varphi }\left|U\right|,\max\left(\left|U\right|\right)\right]K\;1<C_{\varphi }<4$$

(4) $$K=\frac{\nabla \varphi }{\left|\nabla \varphi \right|}$$

The droplet volume fraction (***φ***) can be utilized to gauge the average volume fraction of continuity density and viscosity within each control volume. Similarly, other volume fraction averages characterize functions in an analogous manner.

(5) $$\mu =\varphi \mu_{droplet}+\beta \mu_{air}$$

(6) $$\rho =\varphi \rho_{droplet}+\beta \rho_{air}$$

For both Newtonian and incompressible laminar flows, a simulation is developed based on the instantaneous and three-dimensional laws of conservation of mass and momentum, taking into account the presence of substrate tension and gravity. In this context, P represents the pressure of the fluid mixture, ***ρ*** denotes its density, and ***μ*** indicates its viscosity. The letter U refers to the velocity vector of the fluid. At the interface between the liquid and gas, g symbolizes the gravitational force, while $F_{s}$ represents the surface tension.

(7) $$\frac{\partial U_{x}}{\partial x}+\frac{\partial U_{y}}{\partial y}+\frac{\partial U_{z}}{\partial z}=0$$

The principle of conservation of momentum of a flowing fluid is expressed by the momentum equation. The sum of all the external and internal forces acting on a flow field determines the rate of change of the total momentum. The fluid momentum equation is written as follows:

(8) VariationandConvectionisequalto:Diffusion,InternalandExternalsource

$$Variation:\frac{\partial U}{\partial \tau }$$

Convection:(U.∇)U

$$Internal\;source:-\frac{1}{\rho }\nabla P$$

$$Diffusion:\vartheta \nabla .\left(\nabla U+\nabla U^{T}\right)$$

$$Eternal\;source:g-F_{s}$$

The force at the boundary between the droplet and the surrounding air is taken into account when the value of $F_{s}$ is influenced by surface tension. The equation (9) is used to calculate this term.

(9) $$F_{s}=\sigma \nabla .\left(K\right)\nabla \varphi$$

### 3.2 Mesh convergence

**Fig. 2:**
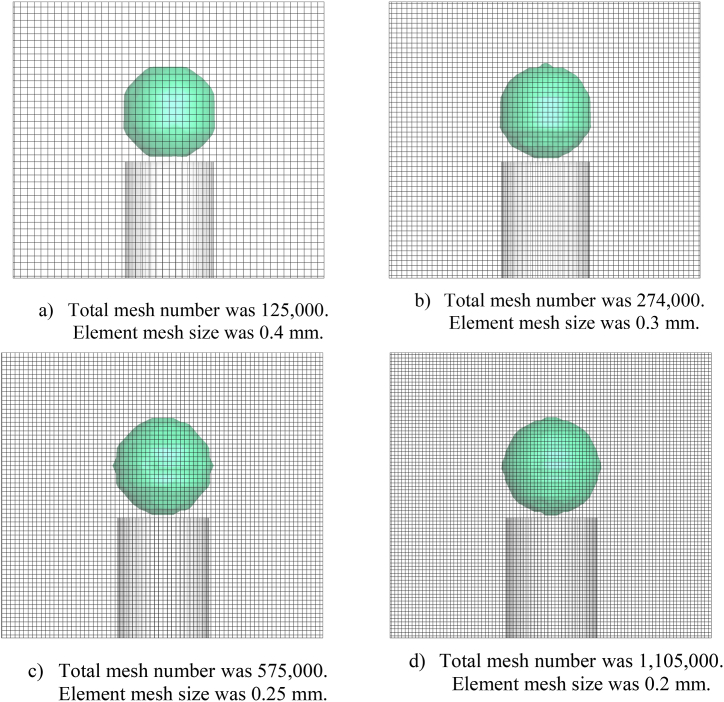
Mesh size comparison for a hollow droplet's form.

Fig. 2 depicts the creation of meshes of Case 1 for various element sizes. The element size was reduced until the Newtonian fluid property did not change significantly, resulting in the optimal element sizing decision for mesh production. The mesh element size was reduced to 0.3 and 0.25 mm at the first 0.4 mm element size chosen and illustrated in Fig. 2a, where the total mesh number was 274,000 the mesh element size was 0.3 mm as shown in Fig. 2b and 575,000 that the mesh element size was 0.25 mm as shown in Fig. 2c. Because element sizes of 0.2 mm and lower have the same Newtonian properties, 0.2 mm was chosen to reduce time and calculation space, with a total mesh number of 1,105,000 was shown in Fig. 2d.

### 3.3 Validation

**Fig. 3:**
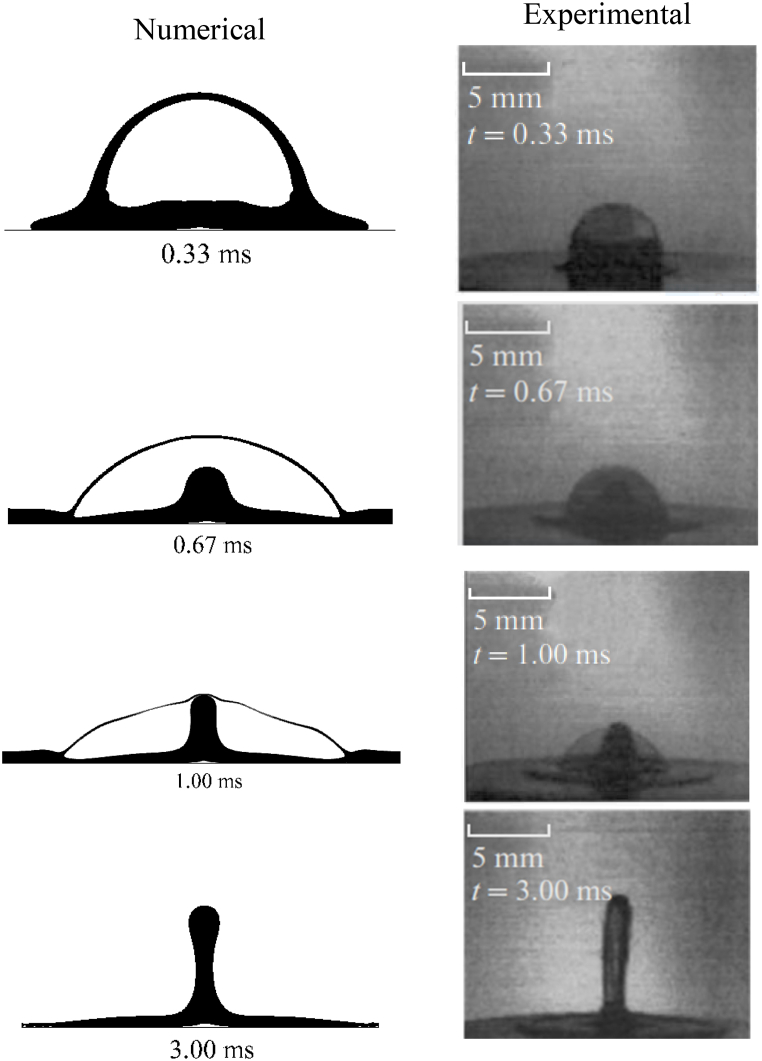
Compared of the effect of hollow droplets between experimental and numerical findings.

**Fig. 4:**
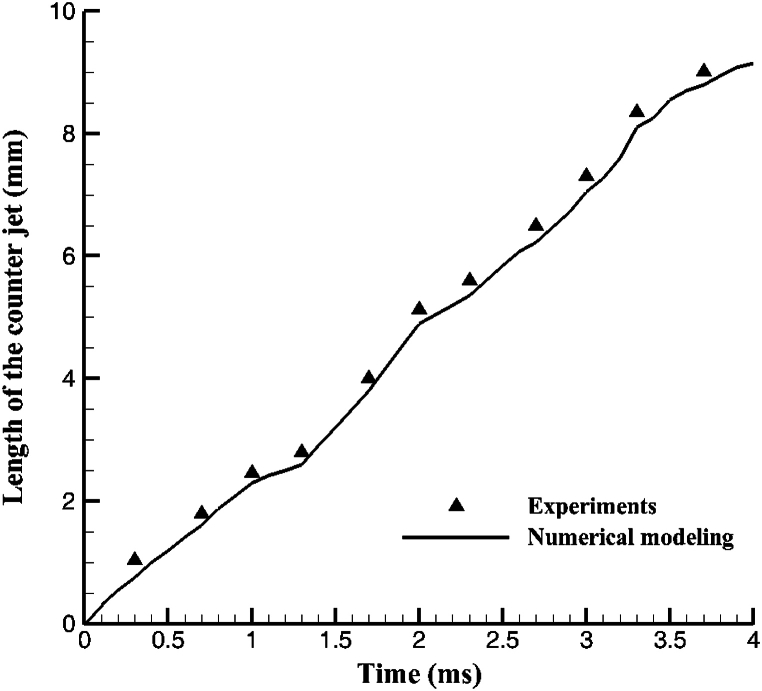
Comparing an experimental and numerical methods to simulate the collision of hollowed drops on a flat, dry surface.

To compare the experimental study results of Gulyaev IP, et al. (2009) [17], The collision of hollow drops on a flat, dry substrate is modeled numerically in three dimensions and is illustrated in Fig. 3. Inside this experiment, a hollow glycerine droplet is slammed at a velocity of 5.94 m/s onto a solid, flat substrate. The issue was modeled utilising Volume Of Fluid (VOF) method utilising the OpenFoam programme to validate our study was shown in Fig. 3 numerical. The task specification details the properties and dimensions of a glycerine droplet. Fig. 4 depicts the morphology of the simulation and experiment with the centre countering jet as well as how an empty drop adheres to a flat substrate.

The counter lifetime was also measured with the Gulyaev figure [17] and compared with the counter lifetime calculated with numerical modeling, as shown in Fig. 4. As a result, the results of the model are different from the experimental results. Due to the limitations of phase interface tracking and image processing, there is a slight difference.

## 4 Results and discussions

### 4.1 Impact process

**Fig. 5:**
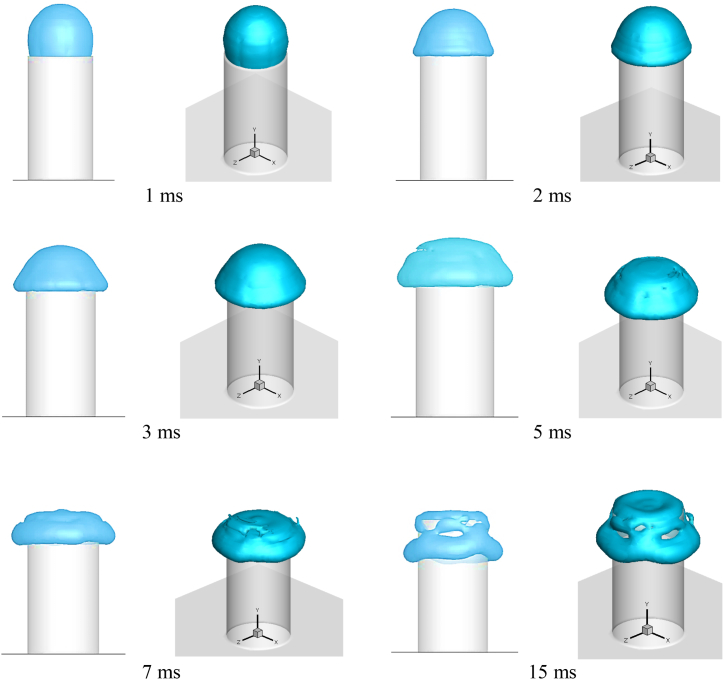
Snapshots of hollow droplets collided on the cylindrical cross-section at various times after impact.

In Case 1, a hollow glycerin droplet collided with the cross-section of a cylinder, and the reaction of contact was recorded at various times after impact (see Fig. 5). Hollow droplets contact the cross-section and spread on the flat surface at a time step 1 ms after impact. The hollow droplet reached the cross section's border 2 ms after impact and began spreading across the cylinder's lateral surface. The gravitational force causes the fluid to spread in the -y-direction on the lateral surface 3 ms after impact. The upper hemispherical shell of a hollow droplet ruptures around 5 ms, causing the droplet to spread further across the surface. At 7 ms after impact, the upper hemispherical shell vanishes completely and is absorbed by the fluid, which spreads throughout the cylinder's lateral surface. As demonstrated in Fig. 5 at 15 ms after contact, some of the fluid of the hollow droplet adheres to the cross-section and the remaining fluid spreads across the letteral surface.

**Fig. 6:**
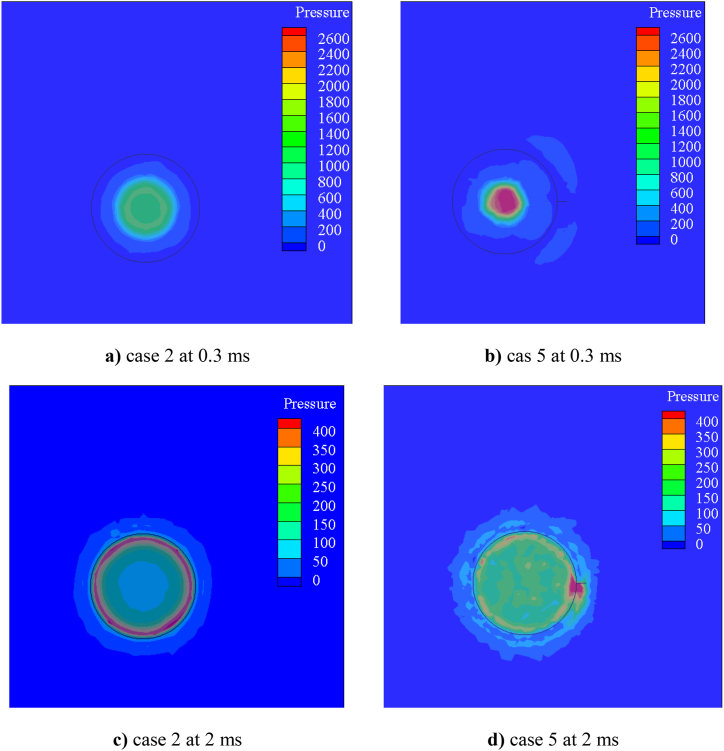
Pressure distribution of hollow droplets collided on the cylindrical cross-section surface at 0.3 ms and 2 ms after impact. In two cases 2 and 5 with 5.25 outer diameter in two shape of simple and spiral respectivly.

The pressure distribution of cases 2 and 5 at two distinct time steps of 0.3 and 2 ms after impact is shown in Fig. 6. At 0.3 ms, a hollow droplet impacts the cross-section of the cylinder, creating a high-pressure zone as seen in Fig. 6a and b. Case 2 has lower pressure than case 5. The spiral caused the pressure zone to shift into the spiral beginning at 2 ms in Fig. 6c and d, but the basic cylinder made a uniform high-pressure gage around the cross-section. The spiral cylinder produces a high-pressure zone and eccentric cross-section by producing a non-uniform space around it. The spiral was added to cylinder and this shape made angle type of cross section. it's made a difference dynamic contact angle, so the pressure distribution was as shown in Fig. 6d.

### 4.2 Morphology of impacting droplet on the simple and spiral cylinders

**Fig. 7:**
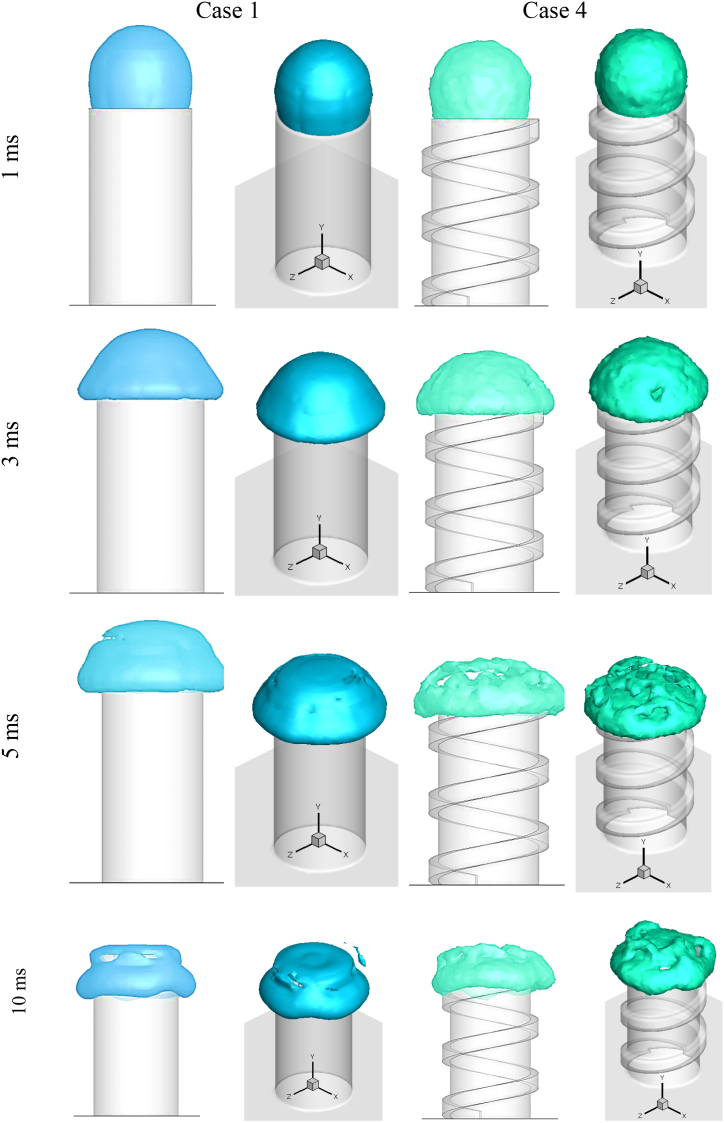
2D and 3D shape of droplet impact on the cylindrical cross-section surface in Case 1 and Case 4 at various times after impact.

Fig. 7 depicts the impact of a drop with an outer diameter of 5 mm on a basic cylinder (Case 1) and a spiral cylinder (Case 4) at various time steps. In Fig. 7, two views of a colliding droplet are shown: XY surface (2D) and 3D view. Hollow droplets impact and spread across the flat surface of the cylinder's cross-section at a time after contact of 1 ms. In Case 1, the droplet reached the cross section's boundary and spread over the letteral surface at time step 3 ms, while in Case 5, the hollow droplet spread non-uniformly due to the spiral, particularly at the start of the spiral, and the upper hemispherical shell ruptured towards the start of the spiral. The upper hemispherical shell ruptures in Case 1 at 5 ms after impact, whereas the hollow droplet entirely ruptures in Case 4. When the hollow droplet is impacted, fluid spread across the cylinder's lateral surface. The spreading of the simple and non-simple cylinders differed in that they were uniform and non-uniform, respectively.

**Fig. 8:**
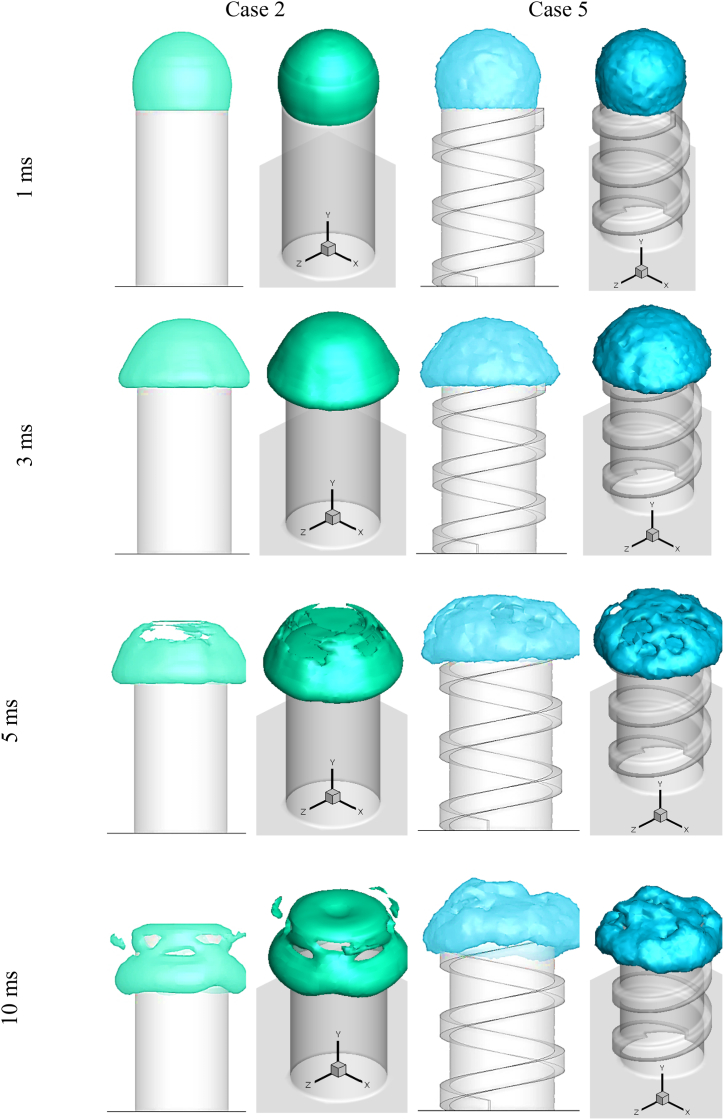
2D and 3D shape of droplet impact on the cylindrical cross-section surface in Case 2 and Case 5 at various times after impact.

Fig. 8 compares the collision of droplet with a 5.25 mm outside diameter on two-cylinder shapes (Case 2 and Case 5). At time step 1 ms, a hollow droplet impacts and spreads across the flat cross-section of a cylinder. At 3 ms after impact, a hollow droplet developed on the cross-section and fell down and spread across the lateral surface in both cylinder shapes. The rupture phenomena occurred in both cases prior to the 5 ms. The fluid of the droplet spreads on the lateral surface 10 ms after impact due to the gravitational force. In Case 2, the splattered fluid was also noticed. In this case, the uniform spreading observed in Case 2 is compared to Case 5.

**Fig. 9:**
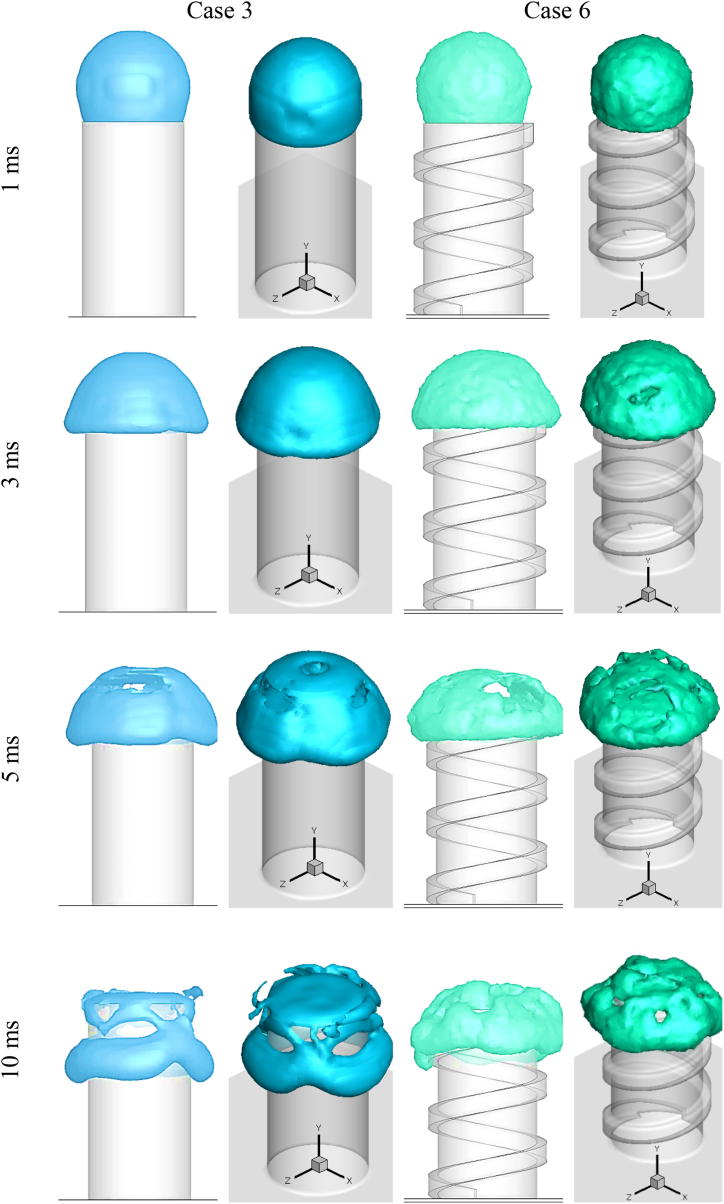
2D and 3D shape of droplet impact on the cylindrical cross-section surface in Case 3 and Case 6 at various times after impact.

The compression between examples 3 and 6, where the outside diameter of the hollow droplet was 5.5 mm, may be shown in Fig. 9. The hollow droplet reached the cross-section of the cylinder's cross-section at time step 1 ms. The fact that this case had a larger diameter than the others is the reason behind this. The hollow droplet distributed uniformly across the lateral surface of the simple cylinder 3 ms after contact, whereas the upper shell ruptured in Case 6. The rupture phenomena occurred in Case 3 at time step 5 ms and progressed in Case 6. In case 3, part of the fluid splashed on the air, and residual fluid spread on the cross-section and lateral surface of the cylinder at 10 ms after impact; however, in case 6, there is no splashed fluid and the fluid spread is non-uniform due to the spiral shape of the lateral surface and low surface tension.

### 4.3 Effects of cylinder shape's and flow characteristics on droplet impact

**Fig. 10:**
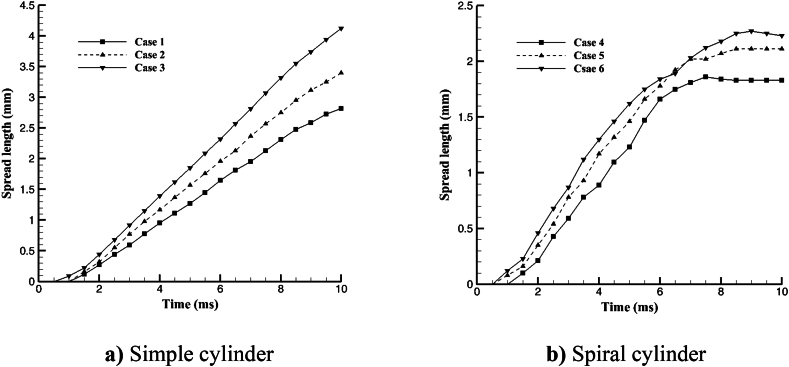
Hollow droplet spread on the lateral surface of the cylinder in –y-direction at the various shape of the cylinder and 10 ms after impact.

In 10 ms after impact, Fig. 10 depicts the length of spreading the fluid on the lateral surface from the cross-section border to the highest amount of fluid spreading on the surface in the -Y direction. The droplet spreading on the simple cylinder including cases 1, 2, and 3 can be seen in Fig. 10a. As can be observed in Fig. 10, the spread length increased as the hollow droplet's outer diameter grew. The maximum quantity of hollow droplet spread length in the -Y direction in a spiral cylinder was measured on the lateral surface. As shown in Fig. 10b, the maximum amount of spread length was observed, and like a basic cylinder, the length rose as the outer diameter increased. The spread length on the simple cylinder rose throughout time, but the spiral cylinder increased for around 8 ms before slowing down or stopping altogether. Fluid adherence to the spiral surface prevents fluid development. The maximum amount of spread length that happened in Case 3 was 4.12 mm and the minimum was 1.83 mm in Case 4.

**Fig. 11:**
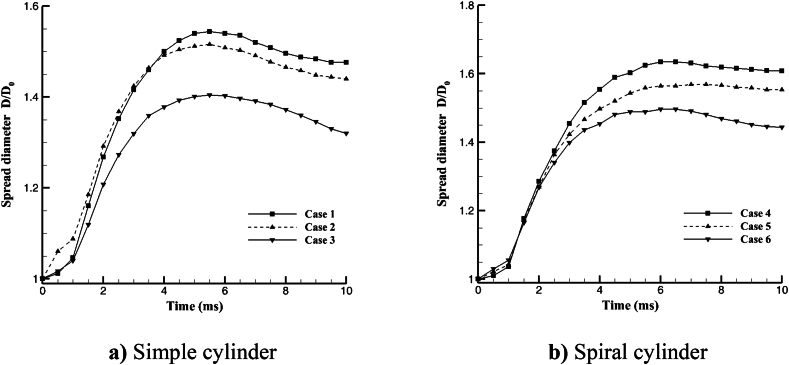
Hollow droplet non-dimensional spread diameter $D/D_{0}$ in +x direction at the various shape of the cylinder and 10 ms after impact.

The non-dimensional spread diameter $D/D_{0}$ is used to calculate the distance between the center of the cross-section and the maximum amount of fluid spread in the +x direction on the ZX plane (see Fig. 11). The outer diameter of the hollow drop before impact was $D_{0}$ and hollow droplet outer diameter after impact was D. The ZX plane was the cylinder's enlarged cross-section surface. The spread diameter on the simple cylinder was depicted in Fig. 11a. The spread diameter increased until the horizontal pressure force was able to overcome the gravitational pull. As a result, before the 5 ms spread, the diameter of the spread rose, but then began to diminish. Because the hollow droplet reached the boundary cross-section earlier than the other cases, Case 3 has the smallest amount. Fig. 11b depicts a spiral cylinder containing cases 4 to 6. The spread diameter expanded with time until it reached 6 ms, at which point it stopped or reduced. When can be observed in the simple cylinder, as the outer diameter increased, the spread diameter shrank. The least amount distributed diameter in Case 3 was 1.404, whereas the highest amount in Case 4 was 1.625.

## 5 Conclusion

Impingement of thin droplet on the solid substrate of the cross-section of the cylinder in the two shapes of lateral surface simple and spiral in this paper. This present study investigated the dynamic behavior and droplet properties. The influence of hollow droplets was studied, and the following are some key points:

• The simple cylinder made a uniform high-pressure gage around the cross-section but The spiral cylinder produces a high-pressure zone and eccentric cross-section by producing a non-uniform space around it.
• Hollow droplets spread on the lateral surface in all cases that uniform spread happened in cases with a simple lateral surface and non-uniform happened in the spiral lateral surface.
• Droplets with a bigger outer diameter have more spread length than droplets with a lower outer diameter. A simple lateral surface has a bigger spread length than a spiral surface. So, observed the maximum length in Case 3 was 4.12 mm, and the minimum was 1.83 mm in Case 4.
• Spread diameter $D/D_{0}$ was assumed on the cross-section plane and the maximum amount in Case 4 was 1.625 and the least amount distributed diameter in Case 3 was 1.404.

Data included in article/supplementary material is referenced in the article.

## CRediT authorship contribution statement

**Hossein Sayyari:** Writing – original draft, Formal analysis. **Mohammad Mohsen Peiravi:** Methodology. **Javad Alinejad:** Methodology.

## Declaration of competing interest

The authors declare that they have no known competing financial interests or personal relationships that could have appeared to influence the work reported in this paper.
